# 中性粒细胞胞外诱捕网与肿瘤转移的研究进展

**DOI:** 10.3779/j.issn.1009-3419.2019.09.08

**Published:** 2019-09-20

**Authors:** 欣伟 张, 勇 王, 勇 李

**Affiliations:** 1 330006 南昌，南昌大学第一附属医院肿瘤科 Department of Medical Oncology, The First Affiliated Hospital of Nanchang University, Nanchang 330006, China; 2 341000 赣州，赣州市人民医院肿瘤科 Department of Medical Oncology, Ganzhou People's Hospital, Ganzhou 341000, China

**Keywords:** 中性粒细胞胞外诱捕网, 中性粒细胞, 肿瘤转移, 循环肿瘤细胞, Neutrophil extracellular traps, Neutrophils, Tumor metastasis, Circulating tumor cell

## Abstract

近年来，研究发现中性粒细胞在多种适当刺激下向细胞外释放一种纤维网络状结构，即中性粒细胞胞外诱捕网（neutrophil extracellular traps, NETs）。在机体中具备捕捉和杀死病原体而参与免疫应答的重要生理作用。目前，越来越多研究证据认为NETs除了可以参与炎症、血栓形成等方面，而且与肿瘤的转移密切相关。肿瘤转移过程由多个步骤构成，机制复杂，是造成癌症患者临床上治疗失败和死亡的主要原因之一。因此，NETs参与肿瘤转移相关机制的研究对肿瘤治疗具有重要意义。本文将对NETs与肿瘤转移及相关机制的研究进展做一综述。

## Abstract (en)

Recently, studies showed that neutrophils extracellular traps (NETs) are the fiber network structures formed by the materials released by neutrophils under various appropriate stimulation. NETs have been indicated to trap and kill microorganisms, playing a critical role in immune responses. It was pointed out that NETs can not only be involved in inflammation and thrombosis, but also is intimately related to tumor metastasis. Therefore, the study of NETs in tumor metastasis is of great significance for tumor therapy and the progress of NETs in tumor metastasis and the relevant mechanisms is summarized.

中性粒细胞是机体非特异性免疫中的主要组成成分，不但能抵制并杀灭微生物和病原体，并且在组织修复中也具有重要作用^[1]^。然而目前研究发现，中性粒细胞能够经过多种途径影响恶性肿瘤的增殖、生长及远处转移^[2]^。肿瘤的远处转移是恶性肿瘤最为突出的一种生理学表现, 也是癌症患者的主要死因。其过程复杂，大致包括肿瘤细胞从原发灶脱离、降解细胞外基质、进入血液循环、发生免疫逃逸、侵犯远处器官等步骤。有观点认为中性粒细胞及其有关成分几乎参与肿瘤进展和转移的每一过程^[3]^，能够分泌体内多种物质影响肿瘤微环境，进而促进肿瘤转移和侵袭^[4-6]^。中性粒细胞在多种刺激因子作用下，向胞外释放称为中性粒细胞胞外诱捕网（neutrophil extracellular traps, NETs）的一种纤维网状结构^[7]^，参与机体非特异性免疫应答。最近NETs的相关研究大量被报道，包括炎症、血栓形成及肿瘤等相关疾病^[8]^。最新研究^[9]^表明NETs除了发挥固有免疫作用外，与肿瘤的进展及转移也关系密切，可以通过多种不同机制参与肿瘤侵袭转移过程。

## 1 NETs的构成及形成机制

Brinkmann等^[7]^在2004年初次发现并报道，活化的中性粒细胞向胞外释放一种网格状结构，即NETs，该结构能够捕捉并阻止多种病原体在宿主内播散，是一种潜在的杀菌机制。NETs是由去聚化的染色质和多种蛋白颗粒构成，除了疏松的DNA骨架外、还有基质金属蛋白酶9（matrix metalloproteinase-9, MMP-9）、中性粒细胞弹性蛋白酶（neutrophil elastase, NE）、组织蛋白酶G（cathepsin G, CG）和髓过氧化物酶等围绕并附着^[7]^。当NETs的DNA骨架被脱氧核糖核酸酶（deoxyribonuclease, DNase）降解时，就几乎丧失了这种胞外杀菌的能力，这表示DNA的纤维骨架是形成NETs不可或缺的重要结构。

NETs由中性粒细胞死亡后释放胞内物质而形成，这种死亡有别于细胞凋亡和坏死，将这种特殊的方式称为NETosis^[10]^。NETosis分为细胞溶解性和自发性（囊泡释放）两种形式。其中细胞溶解性的方式进程发生十分迟缓，需要3 h-4 h。形成过程主要表现为：在相关的适当刺激下，中性粒细胞的细胞核变形、染色质疏松化，核膜与颗粒膜发生分离，最后，NETs由于细胞膜裂解而从细胞内释放到胞外^[10]^。此外，有研究认为除细胞裂解方式外，NETs也可以自发性产生，该过程独特、快速并且不依赖还原型辅酶Ⅱ（NADPH）氧化物。Florian等观察到中性粒细胞可以通过囊泡运输机制排出核DNA，参与NETs的形成，无胞核的中性粒细胞细胞膜完整，仍保留吞噬等生理特性^[11]^。

NETs的形成主要与肽酰基精氨酸脱亚胺酶4（peptidylarginine deimnase 4, PAD4）和活性氧类（reactive oxygen species, ROS）的调节相关。染色质疏松化是NETs构成的最具特色的变化，Wang等^[12]^研究认为，PAD4催化组蛋白中的精氨酸实现瓜氨酸化而导致DNA骨架解螺旋，去聚化的DNA与胞质内蛋白颗粒相融合，形成疏松的NETs纤维网络样结构。Li等^[13]^的研究中除去中性粒细胞的*PAD4*基因的小鼠无法产生NETs，实验组加入野生型中性粒细胞，NETs又能够产生，该研究说明PAD4是调节NETs形成的一个重要因素。Kerlsson等^[14]^证明，佛波酯（phorbol myristate acetate, PMA）加入中性粒细胞中，可导致NADPH氧化产生ROS，进而中性粒细胞释放出细胞核、髓化过氧化物酶等多种NETs的组成成分。慢性肉芽肿病人的NADPH氧化酶发生基因突变，难以形成ROS，而无法激活中性粒细胞死亡途径和形成NETs^[10]^。

## 2 肿瘤转移与NETs

多数研究^[15, 16]^发现，NETs在多种恶性肿瘤中呈现高表达状态。研究^[15]^发现，与健康志愿者相比，结肠癌患者的NETs水平明显更高，并且术前刺激引起NETs增加的患者明显易出现术后并发症，延长住院时间。在尤因肉瘤中，具有高水平的NETs患者在强化化疗后易出现转移和早期复发，预后较差^[17]^。这些结果表明NETs可能作为一个潜在的预后标志物，也提示NETs能够通过某些途径促进肿瘤进展和转移。

肿瘤转移（tumor metastasis）肿瘤细胞脱离原发部位，抵达远处组织或器官继续增殖生长的过程。肿瘤细胞先离开原发部位，进入细胞外基质（extracellular matrix, ECM）并与之粘附，然后释放相关蛋白酶降解ECM穿过ECM和基底膜后进入循环，在循环系统中躲避免疫细胞的监视，随后通过血管壁到达继发部位，在新生血管形成的环境下新的转移灶。肿瘤转移是肿瘤患者病情的恶性进展，对治疗癌症提出了巨大挑战。目前，越来越多的研究证据表明NETs可以促进肿瘤进展和转移^[9, 18, 19]^。NETs通过降解细胞外基质、捕获循环肿瘤细胞、破坏血管完整性、诱导上皮间质转化（epithelial-mesenchymal transition, EMT）及促进血管生成等几个方面影响肿瘤侵袭及远处转移。

### 2.1 NETs降解细胞外基质

ECM网状结构在肿瘤细胞侵袭、进入循环系统过程中相当于一个屏障作用，因此，降解ECM的过程能够有效地促进肿瘤发生转移。研究显示，NETs中存有的蛋白降解酶可以通过溶解ECM的方式来提高肿瘤侵袭和转移的能力。MMP-9是NETs的重要组成成分。多项研究^[20, 21]^表示，MMP-9不只在细胞增殖和凋亡中扮演重要角色，还可以降解ECM成分，为肿瘤细胞迁移做好通道准备，加快了肿瘤细胞转移。金属蛋白酶组织抑制因子（tissue inhibitor of metalloproteinase, TIMP）对MMP具有抑制作用，Aoki等^[22]^研究证明，TIMP通过抑制MMP-9的活性来限制肿瘤细胞降解ECM和侵袭过程。因此，NETs有可能通过其成份MMP-9降解ECM参与肿瘤转移。

研究^[23, 24]^显示，NETs中的NE与金属蛋白酶具有协同作用，并且能够降解蛋白抑制物。Wislez等^[23]^研究中性粒细胞在肺腺癌转移中的作用时发现，NE可以作为中心粒细胞膜的结合分子与癌细胞结合，进而加快肿瘤细胞增殖、生长和远处转移。多数学者认为CG具有降解细胞外基质的作用^[25, 26]^，但目前对于NETs中NE和CG在肿瘤转移的具体作用机制却鲜有报道，仍需更多试验研究去进行深入了解。

### 2.2 NETs捕获循环肿瘤细胞

循环肿瘤细胞（circulating tumor cell, CTC）是指从原发部位或转移灶脱落，进入血液循环而逃脱机体免疫攻击的肿瘤细胞，经血液游走、粘附后定植于远处器官，生长、增殖，逐步形成肿瘤转移灶。研究^[27]^证实CTC在肿瘤转移过程中具有重要临床意义，可以作为多种转移癌症的预后标志物。研究表明，NETs能够通过捕获CTC，从而促进黏附作用、实现免疫逃逸等多个途径参与肿瘤转移机制。

#### 2.2.1 NETs利于CTC黏附

NETs的去聚集状态、立体的DNA纤维状网络能够有效捕获循环中的肿瘤细胞，并帮助CTC锚定在组织，导致肿瘤细胞粘附于组织的数量增加^[19]^，而肿瘤细胞的粘附机制在肿瘤转移途径中极其重要，故NETs可以通过捕获CTC利于其粘附组织而促进转移^[9, 19, 25]^。Cools-Lartigue等^[19]^研究发现，通过荧光共聚焦试验证实并且在扫描电子显微镜下直接观察到，在PMA刺激下，中性粒细胞在A549肺癌细胞周围产生网状DNA结构，NETs的网状DNA结构通过与A549肺癌细胞的细胞膜直接接触，促进肿瘤细胞在组织上粘附从而发生肿瘤转移; 并且这种粘附作用可在DNase作用下被消除。而中性粒细胞并不能直接与肿瘤细胞接触，这提示CTC粘附于组织的机制主要与NETs的网状DNA诱捕功能有关^[19]^。

NETs除了具有促进CTC粘附于组织的作用，也可直接促进CTC对血管壁的粘附。体内动物实验^[28]^表明，NETs捕获肿瘤细胞后使其粘附于血管壁，进一步破坏内皮细胞，可以使血管的渗透性增加，肿瘤细胞突破血管壁抵达远隔器官形成新转移灶。Najmeh等^[29]^研究发现，β1-integrin在介导NETs与CTC相互粘附的过程中具有重要作用。通过构建肺癌小鼠模型模拟术后炎症环境发现，炎症状态下β1-integrin表达上调，肺腺癌细胞粘附NETs的数量明显增加，并且这种效应可通过使用DNase或PAD4抑制剂所消除。其他研究^[30]^也证实，在手术应激状态下，能够诱发机体NETs的产生，促进CTC的粘附和微小转移灶的生长。

#### 2.2.2 NETs促进免疫逃逸

CTC在转移过程中进入血液循环不一定能够存活，免疫系统能够对循环肿瘤细胞监视并进行杀灭，因此，躲避免疫系统监视是肿瘤转移的形成的重要过程。多数研究认为NETs可以促进免疫逃逸^[15, 31]^。在血液循环中，NETs可以网捕并锚定CTC，在免疫细胞和CTC之间形成一道不易穿过的物理屏障^[32]^; 同时，NETs和溶解后相关成分阻止免疫细胞在机体中的生理作用，因此免疫系统对循环中肿瘤细胞的识别和杀死能够被躲避^[25, 32]^。

### 2.3 NETs改变血管完整

血管的完整性的维持和稳固主要依靠于血管的内皮细胞紧密的相互连接，完好的血管壁对肿瘤转移起到了屏障作用。血管内的NETs通过破坏内皮细胞之间的正常连接提高局部血管渗透性，破坏完整性，使肿瘤细胞更易溢出，利于种植到远隔器官并形成微小转移灶^[33]^。Pieterse等^[34]^报道VE-cadherin在参与血管内皮细胞的衔接与稳固具有重要功能，NETs相关蛋白酶通过引起VE-cadherin表达下调进而导致细胞与细胞之间连接完整性破坏，造成血管泄漏，导致大量肿瘤细胞突破血管壁的基底膜流至循环系统。

### 2.4 NETs诱导上皮间质转化

EMT是内皮细胞失去细胞顶端极性而转变成间质细胞表型的过程。EMT在肿瘤转移中具有重要地位，它显著提高了肿瘤细胞在体内转移和侵袭能力。在体内，微循环中的NETs能够刺激血管内皮细胞，促使内皮细胞通过内吞机制将NETs摄入细胞内，胞内的NETs能够使β-catenin的核移位，并导致细胞间连接蛋白VE-cadherin表达下调而促使EMT的发生，同时，这一机制也有利于被NETs捕获的肿瘤细胞向组织内转移^[34]^。另外，NETs诱导EMT过程也依赖于蛋白水解酶的活性。其他研究结果表明，MMP-9表达上调除了可以降解ECM，也能够促进EMT的进程和肿瘤细胞的侵袭作用^[35]^。

### 2.5 NETs促进血管生成

研究^[36]^表明，恶性肿瘤需要了拥有血管生成能力才能发生恶性生长增殖和完成转移。故肿瘤转移灶的顺利产生离不开血管生成。血管内皮生长因子（vascular endothelial growth factor, VEGF）是一种具备多功能的促血管内皮细胞生长因子，能够加快血管内皮移动、提高血管渗透性及促进血管生成等，是参与肿瘤转移的重要因素。研究^[37]^发现，NETs中的多种成分可以通过影响VEGF的表达而促进肿瘤生长、转移和血管生成。Bergers等^[38]^发现在胰腺癌的转基因小鼠中毛细血管的血管开关处于静止状态，实验结果显示MMP-9通过刺激VEGF去激活血管开关，促进血管生成。NETs中也存MMP-9，多数证据表明MMP-9除了可降解ECM，还可进一步影响血管生成和提高肿瘤侵袭能力^[9, 31, 37]^。另外，Wilson等^[26]^认为CG不仅具有降解ECM的功能，而且能够刺激VEGF来促使新生血管产生，使转移灶拥有丰富营养供应而继续生长; 在体内骨髓侵袭模型的小鼠中加入CG抑制剂，与对照组相比，加入CG抑制剂的实验组微血管血管密度明显减少，由此证明CG对血管生成具有促进作用。研究^[37]^发现，NETs的成分NE不仅可以降解蛋白酶抑制物来发挥促转移效应，也能够通过激发VEGF的表达而促进肿瘤生长、侵袭和血管生成等多重作用。NETs中多种蛋白成分对肿瘤细胞的侵袭转移作用仍值得进一步研讨。在手术刺激下，NETs能导致库普弗细胞排出TNF-α、IL-6、CXCL-10等多种细胞因子和趋化因子^[39]^，这些因子都对肿瘤转移具有促进效应。研究^[40]^认为TNF-α在肿瘤微环境中可以促进髓样祖细胞分化成血管内皮细胞，引起血管生成; 不少证据阐明IL-6能通过抑制肿瘤细胞凋亡、刺激血管生成等途径，显著地加快恶性肿瘤的进展并促进其转移^[41]^; CXCL-10不仅是鼻NK/T细胞淋巴瘤中一个自分泌侵袭因子^[42]^，而且在结肠癌的转移中也具有促进作用^[43]^。

## 3 总结

NETs是近年来研究的热点，尤其在肿瘤转移中的作用值得关注，研究表明NETs与肿瘤转移密切相关并具有重要的临床研究价值。NETs能够通过不同机制去参与并促进肿瘤的侵袭与转移途径，但当前相关的实验和研究报道非常有限，仅仅局限在表浅机制的探讨。因此，深入具体作用机制的了解仍有待更进一步研讨与补充。在恶性肿瘤实验中应用脱氧核糖核酸酶或PAD4抑制剂等药物，能够阻止NETs的形成进而消除NETs促进肿瘤转移的效应。因此，深入探究NETs及其抑制剂在肿瘤治疗中的作用具有一定临床研究前景。但是，NETs是否能够成为治疗癌症的新靶点，仍需要大量的实验进行研究和探讨。
